# Dr. Macculloch on the Propagation of Malaria

**Published:** 1828-04-01

**Authors:** 


### *Dr. Macculloch on the Propagation of Malaria.*

In the leading article of our 15th Number, we gave a pretty full account of the first six chapters of Dr. M.'s very interesting work. We now propose to dedicate a short article to an important chapter on the propagation of malaria, by which we shall render to the public a much more comprehensive analysis of Dr. M.'s researches, than has yet been offered in any other periodical publication.

We observed, in our former article, that, as Dr. M. had not travelled much himself, he had evidently made good use of his library, in the study of medical topography. We have since learnt that he is indebted to a gentleman, (who has made a comprehensive survey of the Mediterranean shores) for some very valuable information respecting malaria, and those places where that poison most commonly prevails. This circumstance, instead of detracting from the worth of the volume, very much enhances its value in our eyes, as giving a greater authenticity to certain details, than if they had been gathered from books.

### Chap. VII.—Propagation of Malaria.

Next to a knowledge of those localities which give origin to malaria, is an acquaintance with the laws by which it is propagated. It is properly remarked, that whatever this miasma may be in its simple state, it is only as united with the atmosphere that we know it. It must, indeed, be considered as the very atmosphere itself, where it exists—and its propagation must, therefore, be primarily regulated by those laws which govern the motions of the air. Unfortunately, we do not know much of these laws. The union of malaria with air may be more or less perfect, according to the varying conditions of the latter, as to moisture, and other physical qualities—and it is very clear that its propagation is greatly influenced—perhaps totally suspended, or mightily accelerated, by the ever-varying atmospheric conditions. Though not so capable of becoming durably attached to bodies as the matter of contagion, yet there are not wanting sufficient proofs that malaria is attachable to certain solid substances, as vegetables, and, perhaps, to the soil.

The first fact which arrests our attention in the propagation of malaria, is PROXIMITY. While the atmosphere is quiescent, it may be taken as a general rule, that the place in which malaria is produced, or that which is nearest to it, suffers most. This was probably known to the Romans, from the earliest ages; —and hence it may be, that so many of their ancient towns were situated on hills. Some fatality led to the site of Rome itself, in despite of this knowledge, if it did then exist. Dr. M. descants feelingly on the want of attention to this fundamental fact in the propagation of malaria, by men in all ages, while selecting places for towns, encampments, or single mansions.

"Rome perhaps became too gigantic during its period of ignorance, to be afterwards abandoned or transferred: but there was no apparent reason for perpetuating Calcutta, when, from the very hour almost, of its foundation by Charnock, its destructive situation had been demonstrated. That Holland should have persisted in inhabiting that Batavia which it had studied to render even more poisonous than nature had already done, by the model of its own pestiferous father-land, is a problem which Holland must be allowed to explain as it best can."

Innumerable other examples might be pointed out, of the ignorance or obstinacy of our forefathers, in selecting places, that actually courted the grim king of terrors to visit them more frequently than he otherwise would have done. It is chiefly in military service that the ravages of malaria have been conspicuous, and that the want of knowledge, or the want of inclination to listen to the dictates of experience, has entombed thousands in a premature grave! The writings of Lancisi, Zimmerman, Pringle, Lind, Blane, Jackson, and many others, take away all excuse from some of our modern commanders, for the rash manner of proceeding which they have often adopted, and by which armies have been annihilated, expeditions frustrated, and the best laid political projects counteracted. In Walcheren, we lost 10,000 men, and the Antwerp fleet to boot—when the French army attempted Naples, in 1528, they were reduced from 28,000 to 4000 men, in a few days, by choosing an injudicious encampment at Baix! Ignorance, however, might have been better pleaded 300 years ago than now.

"It was a fortunate discovery in fortification, that a dry ditch was more defensible than a wet one; since the safety and efficiency of a garrison seem never to have entered the minds, even of the Vaubans, the Coehoorns, and the Cormontaignes; though far more intimate, it must be supposed, with Malaria than ourselves."

A whole regiment (says Dr. M.) was incapacitated at Malta, and many of our men destroyed, by persisting to occupy a village which the natives had abandoned. A party of 30 men were successively destroyed, by obstinate attempts to occupy, as a telegraph station, a rocky point in Sicily, between Rasaculmo and Spadafora, although we were warned by the natives of the deadly malaria there prevailing.

"Thus also was our hospital at Port Mahon fixed on the precise spot where it received the whole malaria of that pernicious valley, pestiferous during four months of the year; while by choosing the elevation of Cape Mola, at its north-eastern margin, these bad effects would have been entirely avoided."

The next subject which we are to notice, and which is intimately connected with proximity, is CONDENSATION of malaria. It is natural to suppose, that the gradual production of this poison must occasion a gradual accumulation, unless decomposed or dispersed. Thus, we might anticipate that a marsh, confined within the walls of a forest, as in the pine swamps of America, or the marshy ground of a jungle, or even in our own moist woods, would accumulate malaria, and thereby render it unusually virulent. This is confirmed by experience.

So, also, a marsh, enclosed between high hills, or otherwise deprived of ventilation, is generally very insalubrious.

"How nearly this general rule may be applied to our own country residences, where uniting stagnant or still waters to the confinement of a woody lawn, it is quite superfluous to say. Those who cannot profit by general principles, but who must, at every minute, have the application made for them, are not of a capacity to profit by any thing."

And now for the MIGRATION OR DISPERSION of malaria. If currents of air always moved horizontally, as weather-cocks or the sails of ships would indicate, the matter might be more easily investigated. But the fact is, that atmospheric currents are irregular and intricate in the highest degree—nay further, they scarcely, in any instance, obey the common law of rarefaction, or unequal density, by which they are supposed to be regulated. If, therefore, we cannot explain how a current of malaria may be directed or limited, so, no movement can occur in this poison, however unexpected, that may not find its solution in the capricious currents of the atmosphere. If these currents move vertically upwards, so may malaria—if downwards the malaria may descend. Indeed, both these facts are ascertained. If there be curvilinear courses of malaria, there are curvilinear winds enough to justify such courses. But there are phenomena attendant on the propagation of malaria, which cannot, we fear, be explained, even by the capricious and intricate currents of the atmosphere. One of these is the fact, that a marshy spot of ground will produce disease at some distance, while the inhabitants of the marsh itself will be little affected, or escape altogether.

"In Italy, it has been ascertained that the poisonous exhalations of the Lake Agnano reach as far as the convent of Camaldoli, situated on a high hill at the distance of three miles : this instance further proving that, thus far at least, Malaria can be conveyed by the winds. In France, at Neuville les Dames, above Chatillon on the Indre, and at St. Paul near Villars, both situated on high grounds, there are found as many or more fevers than in the marshes beneath where the Malaria is produced, and the same is generally true all through Bresse in the Lyonnais. Thus also the plain of Trappes near Versailles is affected by the marshes of St. Cyr, though considerably elevated above them.

"I am also informed that a case of this nature occurs in Malta, of a very marked nature ; the Malaria which is produced on the beach beneath a cliff, producing no effect on the spot itself, while it affects, even to occasional abandonment, the village situated above. Many more similar instances might be collected ; but I must be content with adding a few from our own country coming under my own observation, and sufficiently well known to be easily verified.

"At Weymouth, where the back-water, as it is called, produces intermittents, and also autumnal fevers, commonly mistaken for typhus, these diseases scarcely affect the immediate inhabitants of its vicinity, but are found to range along the high grounds above ; and the same, in Cornwall, is true of the vicinity of St. Austle, receiving its Malaria from the marshes of St. Blaisey. If I am not misinformed, it is equally true of the marsh of Marazion in the same county.

"The marshes about Erith in Kent, also, are less injurious to the inhabitants of the lower grounds near them than might be expected ; while their effect on the houses which are situated high on the hill above, is such as, at different times, to have been very severely felt by the inhabitants. The same is true of Northfleet, if my information is correct ; or, the fact as stated is, that at some distance, on the high ridge so well known, agues are more prevalent than below and near the point of the production of the Malaria. If this is not to be explained by the flow of a current, so directed as to escape the low grounds beneath these cliffs and declivities, while it ranges across the hills in contact, I have no solution to offer."

Here our author introduces a statement from Captain Smyth's valuable statistical table of Sicily, which appears to generalize the whole of these facts—and lead to the conclusion that, in nearly an equal number of cases, the higher grounds suffer as much as the lower—the locally healthy as much as those which are the very seats of the malaria!

"In this document, out of seventy-six unhealthy towns and villages enumerated, there are thirty-five situated on hills or declivities; while, from his personal information, I may add that many of them are at considerable distances from the tracts which produce the disease. And I may add one remark as to the theory of this propagation, derived from a writer on the climate of Italy. It is, that the southern winds in that country, propagate along the hills, upwards, that Malaria which the northern or mountain ones do not; such winds, independently of their superior power in producing the pernicious exhalations, tending, from their temperature, to ascend the acclivities, while the other winds, as is easily understood, have the opposite inclination."

Dr. M. makes many interesting remarks on the propagation of malaria along vallies, and on the opposite effects produced, in different places, by the same process—the cutting down or the planting of trees. Thus the cutting down of trees, in some places, would let in a stream of malaria—in others, it would keep it out. Hence the topography of places must be well studied, before we venture on any preventive or corrective measures. Thus a convent at St. Stephano, became unhealthy in consequence of cutting down some trees—and the extirpation of a wood brought on severe fevers at Velletri, during a space of three years, as also happened at Campo Salino, in the Pontine Marshes.

We had occasion, in our first article, to advert to the opinion entertained by many, that Rome was, in ancient times, protected from malaria, by groves that were held sacred. Lancisi remarks that, in later times, there was extirpated near Rome, a forest to the southward, reaching from the heights of Fracasti and Albano, to the Tiber, protecting it from malaria so abundantly generated in that quarter by marshes. Thus, says he, was destruction first let in upon the Campagna. Since that date, if Dr. M's information be correct, a similar proceeding seems to have opened Rome itself, in another quarter, to the malaria of that pernicious land. Dr. M. observes that there was formerly, in a situation interposed between the Campagna and the Porta del Popolo, a wood, cutting off the communication through the north-east winds—and it is, since the destruction of this wood, that the new progress of this pest has been noticed. If this be a fact, it will prove a valuable one, as the Papal government will thus acquire a remedy, as far as this point is concerned, which it has long sought for in vain by drainage.

In whatever way the malaria is generated, or makes its way into the everlasting city, its progress seems to be determinate, if slow:—spreading, as it were, from a fixed point, and making, in every year, a further step, so as gradually to drive the inhabitants before it—as far, at least, as they are opulent, and able to quit their unhealthy habitations. The progress and various windings of this malaria through the streets of Rome, are traced for Dr. M. by some hand, who was evidently well acquainted with the medical topography of that interesting city. Dr. M. speculates on the fate of Rome, in mournful accents; but the malaria may take another direction, or some revolution in the bowels of the earth may procrastinate the fall of this mistress of the world. Dr. M. thinks, and with great probability, that the decreasing population itself, is one cause of the accelerated march of the malaria, since nothing checks the generation of this poison so much as dense population and high cultivation. Among the mysterious circumstances connected with the generation and propagation of malaria is its partiality for one side of a street, and its repugnance to the other side.

"In Rome, it is pointed out, in more places than one, that the Malaria, which must there be transported, not generated, will occupy, even with some permanence, and in some instances also, perennially, one side of a garden or a street, while the opposite one remains exempt. If, in some cases, this is connected with that singular propagation just described, it is an explanation that will not solve every case of the difficulty. They who know Rome, and its tales on that subject, will remember the opposed churches where the porter or janitor on the one side, long and invariably suffering from fever, was cured by the mere transference of his office to the opposite side of the same street; and where, at the same time, the duty had been always as safe as it was invariably dangerous or destructive on the other. This is a circumstance indeed of very frequent occurrence in various parts of Italy, but I will only quote one more instance from that country, out of many, because it is well known to many officers then serving with our army in Sicily. The village, the name of which has escaped me, unless that be Faro, was situated above the Faro of Messina; and while one side of the street was in the highest degree pestiferous, producing mortal fevers among the troops, the opposed one was entirely exempt."

Dr. M. endeavours to throw some light on this apparent mystery, but with very doubtful success. It is probable, he observes, that the matter of malaria is often connected with vapour or mist—nay, that it is conducted and confined by this its vehicle. We find dews, mists, and hoar-frosts often occupying a certain extent, both as to height and depth, reaching, for example, a particular hedge in some valley, and then ceasing by a most definite and sudden line; while also terminating at a particular altitude on the trunks and branches of trees, as if suddenly cut off. This being the case, we may conceive how a malaria, thus united with a mist, may be as defined and local as it actually is found to be, in these singular cases. The following is a remarkable domestic instance of the transportation of this peculiar poison, for the accuracy of which our author pledges himself.

"This is the high road between Chatham and Feversham, involving an extent of about twenty miles; and it is here remarked by the inhabitants, that in every village and town, including also the detached houses, and comprising, from Chatham, Raynham, Newington, Sittingbourne, Bapchild, and Boughton, the ague occurs, on the left hand side of the road, generally, and is unknown on the right side; though the breadth of the road itself forms the only line of separation. If I were to repeat, in addition, some special facts, believed and related by the inhabitants of some of these places, and at Sittingbourne among others, this separation is even more wonderfully and mysteriously precise than the general fact as thus stated would prove it to be. I need only add, that the lands producing this Malaria are situated generally at about a mile distant, on the left hand, being as well known as the road itself."

The above fact rests on such unqestionable authority, and on so many eye-witnesses, that it cannot be doubted. Indeed, it is not much more remarkable than the confinement of malaria to one side of a street. The late terrible epidemic cholera of India, presented innumerable instances still more inexplicable than the above. The cholera would travel for days and weeks along one bank of a small river or even of a nullah, leaving the opposite bank free—and then deserting its favourite track, it would suddenly migrate to the bank that had so long escaped its ravages. For our own parts, we would be more inclined to account for the phenomenon, on the principle of malaria *generated* on the spot, than *transported* to it from a distance. The cause of the epidemic cholera at length reached Cape Comorin, and, from thence, after a certain lapse of time, it travelled to the Mauritius, over a great tract of ocean, and without the aid of a favouring monsoon—nay, against the atmospheric currents. How could this be transportation ? It must have been generation.

The caprices of malaria, in respect to level, are often very difficult of solution. In Italy, where it was first remarked, as a general rule, that the malaria lay near the ground, and was transmitted in the direction of a stratum near its level, in preference to a higher one, the solution was sufficiently obvious. Thus, it was found safe to sleep in the second or upper story of a house, while the fever seized on those below—hence certain popular practices relative to the closing and opening of windows. This fact was also well ascertained in other and distant regions, both in the eastern and western hemisphere.

"The solution here seems easy, and perhaps it is also the true one. It is, that the Malaria is especially united with that transferable substance which forms the foggy stratum ; or that the lowest portion of the atmosphere in the act of depositing water, is its vehicle and its residence."

It is not a little curious that, in some places on the coast of Norfolk, where malaria prevails, the second or upper story of a house becomes its favourite point of attack, while the ground floor invariably escapes. It is probable that, were the circumstances narrowly investigated, there would be found an explanation in the direction of some prevailing winds, or other local incidents, that have hitherto escaped observation.

The poisonous gas constituting malaria, or the vehicle in which it resides, is capable of lodgment and retention in places where it has not been produced. Valetta offers a striking example, in the cases of the Floriana Guard, which suffered so severely, while other portions of the garrison escaped. Here the ditch was very deep and narrow, but so perfectly dry, that it could not be suspected of producing the malaria to which the effects in question were owing. Nor could this be explained, except by supposing that this ditch lodged and protected from dissipation, a current of noxious air, produced from the salt marsh, which seems to be the source of the malaria in Valetta, and which the sea-breeze directed on this spot.

"Nor is this explanation improbable, either for this case or other similar ones, when we know that carbonic acid, as well as watery vapour, or a moist atmosphere, can thus remain at rest on the ground, or in any other place where it is protected from the general circulation of the atmosphere, for a great length of time."

The idea of the attachment of malaria to solid substances is strongly countenanced by many facts. Thus, in the Campagna of Rome, it is remarked that, if the labourers cut down certain plants, (a bushy thistle,) a fever, that otherwise would not have occurred, is the consequence. The malaria is supposed to be entangled within it, and to be let loose by this disturbance. Farther it is observed that, in many parts of Italy, the labourers are safe, so long as they keep to the erect posture; but if they sit or lie down, they are in danger.

"In such cases as this, from the far inferior virulence of the poison with us, the result might be a very slight fever, or at most an ordinary one; while, as such an event would most frequently occur during the time of harvest, it would naturally be attributed to heat or fatigue, or to the influence of the sun; and might thus, under peculiar symptoms, as it most unquestionably often has, be even considered a phrenitis."

In respect to the propagation of malaria, as dependent on certain chemical conditions of the atmosphere, our author has little to offer except analogy, and some detached facts. If not rigidly a gazeous matter, Dr. M. thinks it must be such, or nearly such, in its union with the air. If odoriferous substances be allowed to be analogous, the malarious gas should be most easily united with, and diffused through, a moist atmosphere—as seems to be tolerably well proved to be the case with the matters of contagion generally. A moist atmosphere, indeed, may not only give facilities to the propagation of malaria, but contribute to the production of those diseases which are of a malarious character. But, in how far a moist air is favourable to the primary formation or development of the poison itself, is a question which cannot be solved in the present state of our knowledge. The *propagation* of it by air impregnated with moisture, is, he thinks, pretty well proved, not so much by definite facts, as by a great number and variety of probable circumstances. Thus, the popular opinion goes not only to the belief that malaria is conducted by common fogs, but that these fogs themselves are the poison, or, at least, the cause of the diseases. This is the opinion in Holland, in America, and even in this country. The intermixture of malaria appears to be the real cause of the pernicious nature of fogs, making allowance for the effects of cold and moisture at the same time.

"If it were not so, the same diseases which the pernicious fogs of fenny countries produce, should occur in elevated or mountainous situations subject to be involved in clouds, since the cloud is, in every other respect, a fog. If it were not so, the fogs of dry countries should produce the same diseases as those of moist ones, which they do not; and if it were not so, the westerly fogs that so often arrive in our island from the Atlantic, should generate the diseases of Malaria, like the easterly ones arriving from Holland or formed on our own fenny coasts, which they are never known to do. And to confirm this, it is remarked, that while, in Flanders, (in Artois,) it is the south-westerly and southerly winds which bring and spread disease, in consequence, obviously, of the lands which they traverse, as well as of their own conducting qualities, it disappears as soon as the sea wind from the northern quarters sets in, although this is accompanied by dense and durable fogs. And the same rule will be found to hold good in many parts of the Mediterranean, as well as in France, in numerous cases."

The next fact is analogous, though somewhat different. It is equally a matter of popular belief and medical evidence. It is the pernicious nature of the morning and evening mists formed on low grounds. In the hotter climates, the effects of such mists in generating fever are very notorious—and this fact certainly strengthens the doctrine that the watery or moist atmosphere is the active conductor or repository of malaria—and that when the former is dissipated, as by the sun in the day, the latter is checked in its progress—possibly in its production. The poisonous qualities that have been attributed to dews in hot climates are doubtless owing to their holding miasmata in solution.

"Thus also is it especially remarked, that if a hot day is succeeded by a cold and damp night, the effects of Malaria are much augmented; and the same analogy holds as to similar changes in seasons, or as to incidental ones occurring in any manner. Hence if cold and wet weather should unexpectedly take place in the midst of a hot summer, an augmentation of severity, or a state of disease before not in existence, will occur; and hence also severe epidemics occur particularly, if, to such a hot summer there should succeed a cold and rainy autumn; the production of the poison, as I formerly remarked, being apparently augmented in this manner, while the atmosphere is also rendered a better conductor."

The effects of rains in hot climates are accounted for in the same way by our author, who disagrees with Park and Lind as to the power of rain or rainy seasons to produce intermittents by themselves. He here mentions a curious popular belief, grounded on popular experience, in Italy—namely, that there is no danger from malaria, however plentiful it may be, after nine o'clock at night—in other words, that its influence belongs to evening rather than to night.

"It is conceived, of course, here, that as it is entangled in the morning vapour, becoming dissipated or destroyed as the sun approaches the meridian, so when the condensation of the evening mists has been completed, it is precipitated and rendered inert or null."

This is the explanation which Dr. Johnson gave nearly 20 years ago, with the addition that, as the earth, in hot climates, continued to be hotter than the air for some hours after sun-set, so there continued to be an extrication of miasmata from the earth, for some time after sun-set, thus meeting with, and augmenting the miasmata falling with the dews. In this country, indeed, the night air is considered unhealthy—but this circumstance is generally attributed to the cold of the night. Dr. M. believes this explanation to be quite erroneous, and the following are his sentiments.

"It is thought unwholesome because it is cold, or because it is damp: these are the reasons assigned; but the philosophy is false or confused, and thus the rule of avoidance becomes an inconvenience without being a precaution; while as an inconvenience, it is for ever broken. It is broken also when this air is not damp and not cold, because the philosophy is erroneous: and hence danger and disease which real knowledge would have prevented. No one fears a summer evening, even a mild summer night, unless indeed he shall find or fear a dew. Yet here lies the very danger; in a land of meadows and parks and ponds and rivers and woods, a thousand times more hazardous than all the nights of all the winters that ever were. This is the real night air to be feared, even though the grey mist should not rise, as it is called, or the dew not fall. To take a pleasant evening walk by the banks of the river or the lake, to watch the trout rise from the fish-pond or the canal at the evening flies, to attend the milking of the cows in the green meadow, to saunter among wet groves till the moon rises, listening to the nightingale, these, and more, of such rural amusements and delights, are the true night air, the Malaria, and the fever."

The prevention of such malarious influences in the night season, hinges principally on exercise, and invigorating food and drink. How far smoking and stimulant liquors may be preventive of the operation of miasmata, it is difficult to say, though the general opinion is that they are salutary. To sleep in a miasmal situation, exposed to the night air, is, of course, most dangerous. In this place our author dwells a good deal on *food*, as rendering the body more liable to the impressions of malaria, especially in hot countries, when taken in immoderate quantities, at improper times, and of too animalized nature. It is a fact, that the natives of hot climates almost always breakfast before sun-rise, and dine after sun-set. This is the case, at least, with the great tribes of the Asiatics. They eat but little in the heat of the day, and that chiefly vegetable food. They drink cooling beverages then, and repose as much as possible in the shade. The Europeans too often exercise, eat meat, and drink stimulating potations, throughout the fiery heat of the day, and we all know the rate of mortality among them. It is highly probable, that much of the sickness among Europeans is caused in this way.

It is a curious fact, in the history of malaria, and contrasts strongly with the properties of contagion, that the former is less readily propagated through dense population and dirty streets than along the most spacious terraces, or through the thinly inhabited suburbs. Thus the JUDAICUM at Rome (the Saffron Hill or St. Giles's, of London) escapes the malaria in a remarkable manner:—and so a great number of examples might be cited of the same kind. Perhaps the following explanation may be as good as any.

"The Malaria must be a chemical compound, and therefore decomposable: it is, experimentally, decomposed by fire and smoke, and it is therefore probable, that, amid the unknown mixture which forms the atmosphere of crowded streets or habitations, it is actually destroyed."

The last topic which we shall touch upon is, the real or supposed defence against malaria by means of a veil or canopeum surrounding the head. In Malta and some other places the belief in this measure is universal—hence the popular practice of covering the mouth and nose with a handkerchief, in the morning going out, or in other suspicious circumstances. If dependence can be placed in popular belief or assertions, there is some foundation for this practice.

Our limits have been outstripped, and we must bring our account of Dr. Macculloch's first volume to a close. We are now prepared for the promised volumes on the diseases arising from malaria—and these must afford ample materials for interesting discussions. We shall not fail to give our readers due information of all practical matter brought forward by this industrious author. We are sorry to see some of our cotemporaries endeavouring to turn into ridicule all investigation of the nature, laws, and effects of malaria.* They would better exercise their "TALENTS," of which they have unfortunately TOO MUCH, upon the trickeries, the delinquencies, and the *abuses* of the profession. These are the legitimate objects of satire and criticism—not the patient labours of those who are toiling to draw the veil from some of those obscurities that are perpetually entangling the medical practitioner in the labyrinths of error.

### XI.

### MEDICAL EDUCATION.

* Vide "Death in the Bottle," in the 4th number of the MEDICAL GAZETTE.

